# Circular RNAs in Diabetic Foot Ulcers: A Scoping Review of Clinical, Preclinical, and In Silico Evidence on Diagnostic and Therapeutic Potentials

**DOI:** 10.1002/edm2.70155

**Published:** 2026-01-08

**Authors:** Amir Reza Ghafourian, Masoomeh Hamdi, Atefeh Soltan Mohseni, Maryam Davoudi, Hamid Choobineh, Fariba Nabatchian, Reza Afrisham

**Affiliations:** ^1^ Department of Medical Laboratory Sciences, School of Allied Medical Sciences Tehran University of Medical Sciences Tehran Iran; ^2^ Students' Scientific Research Center Tehran University of Medical Sciences Tehran Iran

**Keywords:** angiogenesis, biomarker, circular RNA, diabetic foot ulcer, exosomal therapy, in silico network, non‐coding RNA, wound healing

## Abstract

**Background and Objective:**

Diabetic foot ulcers (DFUs) involve chronic inflammation, impaired angiogenesis, oxidative stress, and disrupted fibroblast–keratinocyte interactions. Circular RNAs (circRNAs), a category of stable non‐coding RNAs, have become essential regulators of these processes; nevertheless, their comprehensive functions in DFUs are still inadequately characterised. This scoping review integrated clinical, preclinical, and in silico evidence on circRNAs in DFUs to assess their diagnostic, mechanistic, and therapeutic potential.

**Methods:**

Systematic searches of MEDLINE, EMBASE, Web of Science, and Google Scholar were conducted on July 16, 2025, according to the PRISMA‐ScR guidelines. Eligible papers included clinical investigations of circRNAs in the tissues of patients with DFUs, preclinical animal models assessing circRNA‐based therapies, and computational predictions of circRNA‐miRNA‐mRNA networks. Information was collected on circRNA expression, molecular targets, clinical associations, and therapeutic effects.

**Results:**

Twenty‐two studies (7 clinical, 13 preclinical, 2 in silico) were selected. Clinical studies found *hsa_circ_PRKDC, hsa_circ_072697, hsa_circ_080968*, and *hsa_circ_0000907* to be associated with wound severity, tissue perfusion, and keratinocyte proliferation in patients with diabetic foot ulcers. Preclinical studies showed that delivery of *mmu_circHIPK3, mmu_circMYO9B,* and *mmu_circ_Astn1* via exosomes or nanoparticles improved angiogenesis, epithelial regeneration, and wound closure. However, the silence of *mmu_circ_0005654* reduced ferroptosis and inflammation. In silico analyses identified potential regulatory axes, such as *hsa_circ_0089761/miR‐146a‐5p/SMAD4* (*Mothers against decapentaplegic homologue 4*) and *hsa_circ_0049271/miR‐24‐3p/JUNB*, that were associated with inflammatory‐angiogenic pathways in this disease.

**Conclusions:**

CircRNAs hold promise for the diagnosis and treatment of DFUs by modulating angiogenesis, inflammation, oxidative stress, and epithelial repair. Standard network‐guided therapies are essential to translate circRNA‐based strategies into clinical practice.

## Graphical abstract

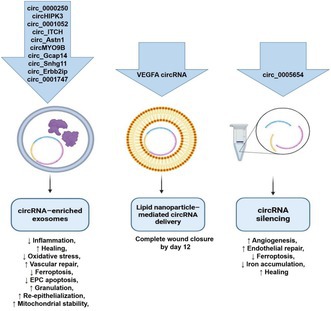

**Abbreviations**
ABI: ankle‐brachial index
ADSC: adipose‐derived stem cell
AGEs: advanced glycation end products
AMPK: AMP‐activated protein kinase
AOPPs: advanced oxidation protein products
AUC: area under the curve
BCL2: B‐cell lymphoma 2
CBL: casitas B‐lineage lymphoma proto‐oncogene
CCND1: cyclin D1
CHIP: carboxyl‐terminus of Hsp70‐interacting protein
circRNA: circular RNA
CRP: C‐reactive protein
DFU: diabetic foot ulcer
DM: diabetes mellitus
ECM: extracellular matrix
EGFR: epidermal growth factor receptor
EPC: endothelial progenitor cell
ERK: extracellular signal‐regulated kinase
FBN1: fibrillin 1
FGF4: fibroblast growth factor 4
FN: fibronectin
FOXO1: Forkhead box O1
GO: gene ontology
GPX4: glutathione peroxidase 4
HDAC: histone deacetylase
HIF‐1α: hypoxia‐inducible factor 1 alpha
Hsp70: heat shock protein 70
HUVEC: human umbilical vein endothelial cell
IGF2BP2: insulin‐like growth factor 2 mRNA‐binding protein 2
IL: interleukin
IRAK4: interleukin‐1 receptor‐associated kinase 4
JAK: Janus kinase
JUNB: Jun B proto‐oncogene
KDM1A: lysine‐specific demethylase 1A
KRAS: Kirsten rat sarcoma viral oncogene homologue
LC3: microtubule‐associated protein 1A/1B‐light chain 3
LNP: lipid nanoparticle
LTBP‐1: latent transforming growth factor beta‐binding protein 1
MDA: malondialdehyde
MGO: methylglyoxal
miRNA: MicroRNA
MMP: matrix metalloproteinase
mRNA: messenger RNA
MSC: mesenchymal stem cell
Nrf: nuclear factor erythroid 2–related factor
PHD: prolyl hydroxylase domain
PI3K: phosphatidylinositol 3‐kinase
PKC: protein kinase C
p‐p38: phosphorylated p38 mitogen‐activated protein kinase
RBP: RNA‐binding protein
RCT: randomised clinical trial
ROC: receiver operating characteristic
ROS: reactive oxygen species
SDF‐1α: stromal cell‐derived factor 1‐alpha
SIRT: sirtuin
SLC7A11: solute carrier family 7 member 11
SMAD4: mothers against decapentaplegic homologue 4
SP1: specificity protein 1
STAT: signal transducer and activator of transcription
STZ: streptozotocin
SUFU: suppressor of fused homologue
TcPO2: transcutaneous oxygen tension (TcPO_2_)
TGF‐β1: transforming growth factor beta 1
TNF‐α: tumour necrosis factor alpha
TSP‐2: thrombospondin‐2
VEGF‐A: vascular endothelial growth factor A
VEGFR‐1: vascular endothelial growth factor receptor 1

## 1 Introduction

Diabetic foot ulcers (DFUs) are among the most disabling consequences of diabetes mellitus (DM), defined by non‐healing ulcers that extend into the subdermis and ankle joint [1]. Globally, DFUs affect approximately 19%–34% of diabetic patients, equivalent to nearly 18.6 million people, of whom 50%–60% develop wound infections and approximately 20% result in lower limb amputation [2, 3]. The mortality rate for people with DFUs is 231 deaths per 1000 person‐years, which is much higher than the rate for people with diabetes who do not suffer from DFUs [3]. These numbers reveal that DFUs are a huge public health issue that makes life worse for the sufferers.

These ulcers arise from a complex interaction of factors, including neuropathy, ischemia, metabolic disorders, impaired angiogenesis, and chronic inflammation [1, 4, 5]. Hyperglycemia disrupts endothelial homeostasis, reduces perfusion, and induces ischemic injury through activation of protein kinase C (PKC) [6, 7]. Neuropathy causes sensory and motor impairments in patients, leading to altered foot and wound biomechanics [8]. In addition, hyperglycemia‐induced immune dysfunction delays wound healing by impairing neutrophil activity, inducing advanced glycation end products (AGEs), and slowing extracellular matrix (ECM) remodelling [1, 4, 5]. Although these mechanisms are well documented, the molecular regulatory networks that impair healing in DFUs are still poorly understood. Furthermore, traditional biomarkers, such as inflammatory proteins (procalcitonin, pentraxin 3, interleukins (IL‐6, IL‐18, IL‐20, IL‐22, IL‐24), and C‐reactive protein (CRP)), metabolic markers (arginine, leucine, and isoleucine), and microbial profiles (gram‐positive cocci and anaerobic pathogens), lack sufficient sensitivity or specificity for early diagnosis or monitoring of treatment responses to this disease [9, 10, 11]. Therefore, the identification of novel molecular signatures with diagnostic and therapeutic potential is a key unmet need in the management of DFU.

Circular RNAs (circRNAs) are a recently discovered class of non‐coding RNAs. These molecules form covalently closed loops that make them highly resistant to exonucleases [10, 11]. These molecules generally act as sponges for microRNAs (miRNAs), regulate mRNA stability and transcription, and interact with RNA‐binding proteins (RBPs) to influence post‐transcriptional gene expression. Recent studies have demonstrated their importance in processes involved in wound healing, including angiogenesis, inflammation, oxidative stress, and apoptosis [10, 11]. In addition to diabetes, circRNAs have been implicated in many other multifactorial diseases, such as cancer, cardiovascular disease, and neuropathy. This finding suggests that these genetic molecules can participate in the regulation and manipulation of diseases by forming regulatory circRNA‐miRNA‐mRNA networks [12, 13, 14, 15]. Therefore, they may be good targets for the diagnosis, monitoring, and treatment of diseases, especially since they are highly stable and have tissue‐specific expression [10, 11, 16]. However, despite the large amount of experimental and computational data in this area, we still do not know exactly how circRNAs function as biomarkers and therapeutic targets for DFUs.

### 1.1 Rationale and Objectives

Because DFUs have a high incidence of morbidity and death, with rates of 5% at 1 year and 42% at 5 years [17], it is very important to provide trustworthy diagnostic tools and effective treatment plans for them. In this context, circRNAs may help us learn more about the molecular causes of DFUs and provide us with chances to diagnose them early and treat them quickly and effectively. Therefore, this scoping review brought together all the information from clinical, preclinical, and in silico investigations on circRNAs in this field. The goal was to (i) find circRNAs that can be used to diagnose or predict diabetes, (ii) explain how they work in diabetic wound healing, and (iii) point out possible circRNA‐based therapy targets for future study.

## 2 Methodology

### 2.1 Protocol and Registration

This scoping review was conducted in accordance with the PRISMA Extension for Scoping Reviews (PRISMA‐ScR) [18]. The review protocol followed five key stages: (i) identifying the research question, (ii) identifying relevant studies, (iii) study selection, (iv) charting the data, and (v) collating, summarising, and reporting the results. The study protocol was prospectively registered in the International Prospective Register of Systematic Reviews (PROSPERO) under the registration number of CRD42024586997 (https://www.crd.york.ac.uk/prospero/display_record.php?RecordID=586997).

### 2.2 Research Question

The present scoping review aimed to investigate the significance of circRNAs as diagnostic/differentiation (in clinical studies), therapeutic targets (in preclinical studies), and candidate discovery (in in silico studies).

### 2.3 Search Strategy and Databases

A systematic literature search was performed on July 16, 2025, across MEDLINE (via PubMed), EMBASE, ISI Web of Science, and Google Scholar databases. The search was not restricted by publication date to ensure inclusivity of all relevant studies. Both MeSH and non‐MeSH keywords were used in various Boolean combinations. The detailed search strategies for each database are provided in Table 1. To ensure comprehensiveness, the reference lists of all included studies and relevant reviews were manually screened for additional eligible articles. Duplicate records were removed using EndNote 21, and screening was independently performed by two reviewers according to the following inclusion and exclusion criteria.

**TABLE 1:** Search strategy.

Database	Search query
PubMed	((((RNA, circular[MeSH Te) OR (circRNA*[Title/Abstract])) OR (Circular RNA*[Title/Abstract])) OR (closed circular RNA*[Title/Abstract])) AND ((((((diabetic foot[MeSH Terms]) OR (diabetic foot ulcer*[Title/Abstract])) OR (diabetic foot[Title/Abstract])) OR (diabetic feet[Title/Abstract])) OR (foot ulcer*[Title/Abstract])) OR (DFUs[Title/Abstract]))
ISI Web of Science	((TS = (circRNA*)) OR TS = (Circular RNA*)) OR TS = (closed circular RNA*) AND ((((TS = (diabetic foot ulcer*)) OR TS = (diabetic foot)) OR TS = (diabetic feet)) OR TS = (foot ulcer*)) OR TS = (DFUs)
Embase	((TITLE‐ABS‐KEY (circrna*) OR TITLE‐ABS‐KEY (circular AND rna*) OR TITLE‐ABS‐KEY (closed AND circular AND rna*))) AND ((TITLE‐ABS‐KEY (foot AND ulcer*) OR TITLE‐ABS‐KEY (dfus) OR TITLE‐ABS‐KEY (diabetic AND foot AND ulcer*) OR TITLE‐ABS‐KEY (diabetic AND feet) OR TITLE‐ABS‐KEY (diabetic AND foot)))

### 2.4 Eligibility Criteria

The target population consisted of human patients with DFUs and/or experimental mice or rat models of DFU. Eligible clinical and preclinical studies assessed circRNAs in wound tissues or serum profiling. In silico studies were also included if they reported circRNA‐miRNA‐mRNA regulatory networks. Comparators consisted of healthy or non‐DFUs, untreated or treated animal models of DFU, or computational controls obtained from publicly accessible transcriptome datasets. The outcomes of interest encompassed the diagnostic, mechanistic, and therapeutic functions of circRNAs. Acceptable study designs encompassed observational and experimental clinical studies evaluating circRNA expression or function in DFU patients, preclinical animal studies examining targeted circRNA interventions, and in silico studies delineating regulatory networks pertinent to DFU pathogenesis.

We excluded studies that were irrelevant to both DFUs and circRNAs. Studies were also excluded if they did not include primary data (e.g., reviews, comments, or editorials). This thorough approach ensured that research that may shed light on the function of circRNAs in DFUs was all included.

### 2.5 Data Extraction

A standardised data extraction form was developed in Microsoft Excel, pilot‐tested on three randomly selected studies, and refined before final data collection. The following data were extracted:

− *Bibliographic details*: First author, year of publication, country of origin.
− *CircRNA characteristics*: corresponding miRNAs, expression trends, molecular pathways, and functional roles.
− *Clinical study parameters*: Study design, patient population, sample size, DFU grade, and inclusion/exclusion criteria.
− In vivo *experimental details*: Animal model characteristics, diabetes induction method, ulcer creation protocol, therapeutic interventions, and wound‐healing outcomes.
− *In silico dataset features*: GEO accession numbers, dataset types, differential expression analyses, and predicted molecular interaction networks.

### 2.6 Data Charting and Synthesis

Data were charted independently by three authors using a standardised template. Any disparities were resolved by consulting with a supervisor. No meta‐analysis or bias assessment was performed due to methodological heterogeneity. To ensure methodological clarity, clinical, preclinical, and in silico data were extracted and summarised separately in Tables 2, 3, 4. Studies that initially evaluated circRNA expression using the Gene Expression Omnibus (GEO) datasets and subsequently performed validation in human clinical samples were presented in the clinical section (Section 3.2). In contrast, studies limited to bioinformatic analyses without experimental or clinical verification were reported separately in the in silico section (Section 3.4).

**TABLE 2:** Summary of CircRNAs with diagnostic and differentiation roles identified in clinical studies of DFUs (all studies were conducted on wound tissue samples, and wherever other samples were used, this is noted).

No.	Authors (year; country)	Study design/Data source	Study groups (n)	DFU inclusion/Exclusion criteria	circRNA regulation in DFUs	miRNAs	Roles/Mechanistic pathways	Key findings (expression and diagnostic insights)
1	Wang et al. (2020; China) [19]	Experimental case–control study with in vitro functional validation	DFU (*n* = 19) vs. NHW (*n* = 19) vs. intact skin (*n* = 13)	*Exclusion*: antibiotic use, immunosuppression *Inclusion*: chronic (> 2 months), non‐healing, antibiotic‐free	*↑ hsa_circ_0084443* (*circ_PRKDC*)	NA	Regulates JAK/STAT, EGFR, PI3K, ERK, and HIF‐1 pathways; affects keratinocyte proliferation/motility.	↓ in NHW vs. intact skin; ↑ in DFU vs. NHW Potential biomarker for wound chronicity
2	Fu et al. (2023; China)^a^ [20]	Bioinformatics‐integrated clinical experimental study (GEO source: GSE114248)	DFU (*n* = 38) vs. NHW (*n* = 27) vs. DM without DFU (*n* = 15)	*Exclusion*: antibiotic use, immunosuppression *Inclusion*: chronic (> 2 months), non‐healing, antibiotic‐free	*↑ hsa_circRNA_080968, hsa_circRNA_081069, hsa_circRNA_100,980*	*miR‐326* and *miR‐766‐3p* (possible targets of *circRNA‐080968*)	*circRNA_080968* inhibits keratinocyte migration; promotes proliferation	↑ *circRNA_080968/081069/100980* in DFU vs. NHW; ↑ circRNA_080968 in DFU vs. DM ↓ migration and ↑ proliferation of keratinocytes
3	Tian et al. (2023; China) [21]	Clinical case–control study with in vitro mechanistic validation (experimental functional study)	DFU (*n* = 8) vs. NHW (*n* = 8)	*Exclusion*: Drug allergy history, pacemaker, and febrile disease *Inclusion*: > 18 years, DM > 10 years, Wagner grade 2–3, HbA1c ≥ 7.5%	↑ *Hsa_circRNA_072697*	*miR‐3150a‐3p* → *KDM2A*	*circRNA_072697* sponges *miR‐3150a‐3p* → ↑ *KDM2A* → ↓ keratinocyte proliferation/migration Impacts MAPK pathway	Its silencing restores proliferation/migration in AGEs‐treated HaCaT cells
4	Tian et al. (2020; China)^b^ [22]	Bioinformatics‐integrated clinical experimental study (GEO source: GSE114248)	DFU (*n* = 5) vs. NHW (*n* = 5)	NA	*↑ Hsa_circRNA_072697, Hsa_circRNA_405463*	*miR‐3150a‐3p*	Regulates KRAS/MAPK pathway	They proposed as novel DFU biomarkers
5	Chen et al. (2020; China)^b^ [23]	Bioinformatics‐integrated clinical experimental study (GEO source: GSE114248)	Discovery cohort: 10 pairs of DFU and NHW tissues analysed by qRT‐PCR for validation of bioinformatics results Validation cohort 1: DFU (*n* = 20), non‐DFU diabetic controls (*n* = 20), and NHW (*n* = 20); samples included tissue, serum, and serum‐derived exosomes Validation cohort 2 (large‐scale diagnostic validation): DFU (*n* = 65), non‐DFU diabetic controls (*n* = 65), and NHW (*n* = 70)	*Exclusion*: malignancy, hepatic/renal disease, autoimmunity, hypertension, gestational/T1DM, systemic inflammation *Inclusion*: Wagner grade 0–2	*Hsa_circ_0000907, hsa_circ_0057362*	NA	Serum circRNAs negatively correlate with ABI and TcPO_2_ in the patients	Both serum and exosomal *hsa_circ_0000907* and *hsa_circ_0057362* were proposed as novel DFU biomarkers (Serum AUCs: 0.9389 and 0.8792; Exosomal AUCs: 0.8783 and 0.8481)
6	Han et al. (2021; China) [24]	In vitro mechanistic validation study (a follow‐ups study for Wang et al.'s findings on the *circ_PRKDC* upregulation in DFUs [19])	Healthy volunteers with wounds (*n* = 7; 6 mm) vs. unwounded skin (more characteristics of the subjects were not available)	*Exclusion*: diabetes, skin/cardiac disease, infection, coagulopathy, immune disorder, ongoing therapy *Inclusion*: Healthy volunteers	↓ *Hsa_circ_PRKDC* in healthy wounds (Day 1 & 7) vs. intact skin	*miR‐31*	*Hsa_circ‐PRKDC/miR‐31/FBN1* axis	hsa_circ‐PRKDC knockdown → ↓ miR‐31 → ↑ MMP‐2, MMP‐9 → enhanced keratinocyte migration
7	Bai et al. (2025; China)^a^ [25]	In vitro experimental mechanistic study (based on high‐throughput sequencing and functional validation in HUVECs; GEO assigned: GSE286165)	DFU (*n* = 3) vs. normal open foot trauma (*n* = 3)	*Exclusion*: systemic infection, malignancy, Charcot arthropathy *Inclusion*: T2DM, age > 18, Wagner grade 3, 0.5 < ABI < 0.9, ulcer > 1 cm^2^	↑ *Hsa_HLA_DRB1*	*miR‐12,118*	*hsa_HLA_DRB1/miR‐12,118/FLT‐1* (*VEGFR‐1*) axis	Mediates inflammation and angiogenesis

^a^ This study initially conducted a bioinformatics analysis, followed by validation using human tissue samples to confirm the in silico findings.

^b^ The hub genes identified through in silico analysis were further validated using RT‐PCR experiments.

## 3 Results

### 3.1 Systematic Search Results

**FIGURE 1:**
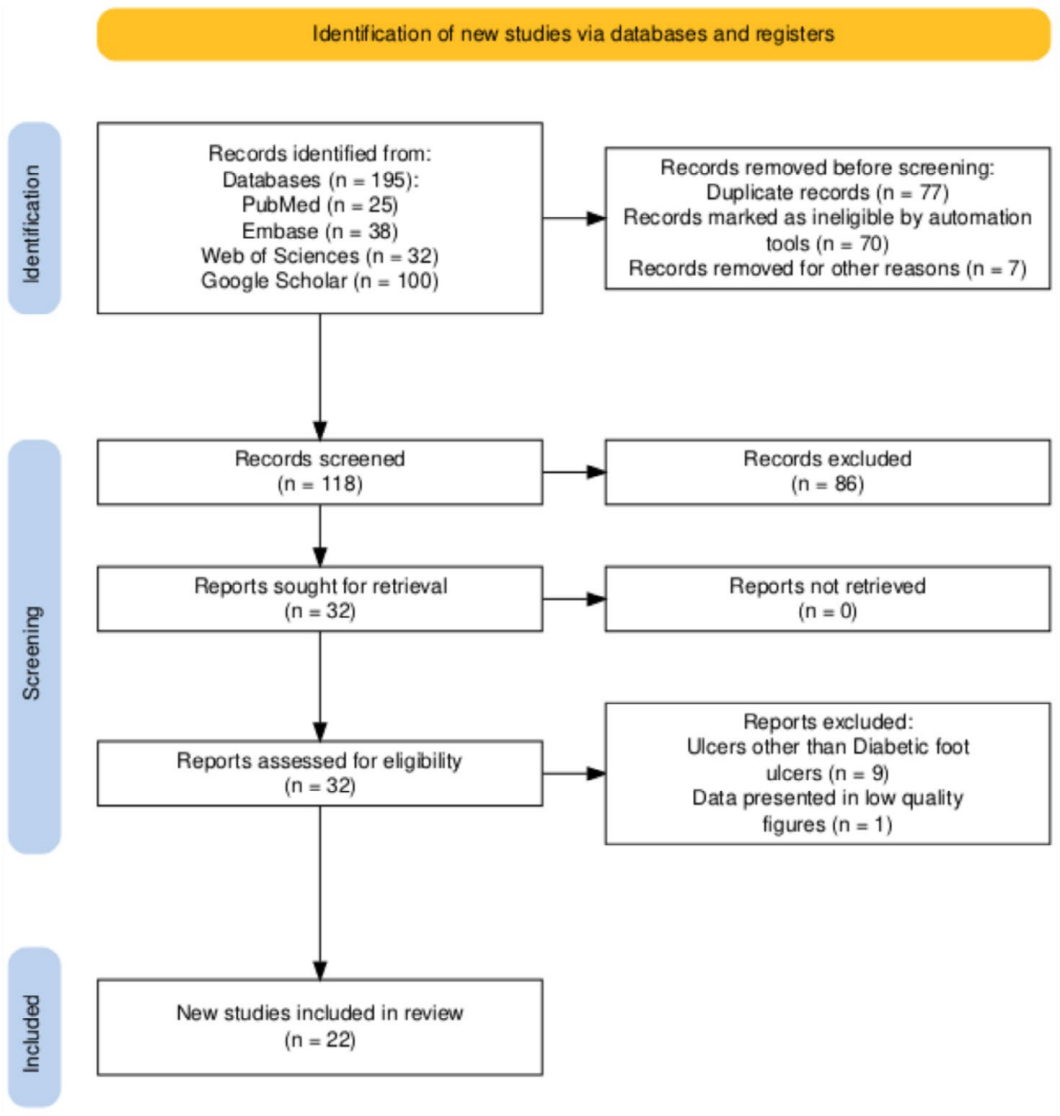
A graphical illustration detailing the processes involved in selecting literature.

A total of 195 records were retrieved from PubMed, Scopus, Embase, and Web of Science. After removing 77 duplicates, 118 studies were screened by title and abstract, of which 32 were subjected to full‐text assessment. Following the inclusion and exclusion criteria, 22 studies were included in this scoping review: 7 clinical, 13 preclinical, and 2 in silico. Details of the screening and selection process are provided in Figure 1.

### 3.2 Clinical Findings (Diagnostic and Differentiation Roles of circRNAs)

Seven clinical trials published from 2020 to 2025 were identified (Table 2) [19, 20, 21, 22, 23, 24, 25]. We aimed to characterise circRNAs in diverse biological samples, including serum, tissue biopsy, and exosomal samples. Different studies used a variety of profiling techniques, including high‐throughput sequencing, microarray screening, and quantitative RT‐PCR validation. Studies varied in terms of inclusion criteria and control group selection, and most compared DFU tissues with healthy or non‐DFU diabetic wound tissues [19, 20, 21, 22, 23, 24, 25].

Wang et al. [19] found that *hsa_circ_0084443* (*circ_PRKDC*) was a crucial transcript that was expressed differently in patients with DFUs. Functionally, *circ_PRKDC* was discovered to influence keratinocyte proliferation and migration by modulating key signalling pathways, including Janus kinase (JAK)/Signal transducer and activator of transcription (STAT), Epidermal growth factor receptor (EGFR), Phosphatidylinositol 3‐kinase (PI3K), Extracellular signal‐regulated kinase (ERK), and HIF‐1. Expression of *circ_PRKDC* was lower in normal wounds compared with intact skin but significantly elevated in DFUs. This data indicated its involvement in compromised wound healing and persistent ulceration [19]. Similarly, Han et al. [24] also validated the regulatory function of *circ_PRKDC* in epidermal regeneration. Thus, inhibiting *circ_PRKDC* facilitated wound healing by reducing *miR‐31* levels, which subsequently elevated the production of Matrix metalloproteinases 2 & 9 (*MMP‐2* and *MMP‐9*). This mechanism made keratinocytes migrate better. Furthermore, *miR‐31* was shown to target *Fibrillin 1* (*FBN1*). In fact, overexpressing *FBN1* largely undid the effects of knocking down *circ_PRKDC*. The dynamic modulation of *circ_PRKDC* throughout the phases of wound healing (downregulated on Days 1 and 7 relative to undamaged skin) indicated its role in the temporal coordination of wound closure [24].

Tian et al. [21, 22] conducted two complementary studies to demonstrate increased levels of circRNA_072697 in DFU tissues. The findings indicate that *circ_072697* acted as a molecular sponge for *miR‐3150a‐3p*. This event led to the overexpression of *lysine demethylase 2A* (*KDM2A*) to stop keratinocyte growth and movement. Their previous bioinformatics study (2020; GSE114248 dataset) had already linked this mechanism to the Kirsten rat sarcoma viral oncogene homologue (KRAS)/mitogen‐activated protein kinase (MAPK) signalling cascade [21, 22]. Fu et al. [20] looked at the same GEO dataset again and showed that *circRNA_080968*, *circRNA_081069*, and *circRNA_100,980* were all higher in DFU tissues than in NHWs and non‐DFU diabetic controls. Functional predictions indicated that *circRNA_080968* interacted with *miR‐326* and *miR‐766‐3p*. This function, in turn, reduced keratinocyte migration while stimulating cell proliferation [20].

Chen et al. [23] also combined clinical validation and data mining of the GEO dataset to evaluate circulating circRNAs in DFUs. Two transcripts, *hsa_circ‐0000907* and *hsa_circ‐0057362*, exhibited considerable dysregulation in DFU tissues and serum. Interestingly, they demonstrated negative relationships with the ankle‐brachial index (ABI) and transcutaneous oxygen tension (TcPO_2_), both clinical indicators of tissue perfusion. These results underscored their clinical diagnostic significance and viability as serum or exosomal biomarkers for the noninvasive evaluation of DFUs [23]. Bai et al. [25] recently identified hsa_HLA_DRB1 as a DFU‐associated circRNA by several prediction pipelines, including Find_circ, CircRNA_finder, CIRCexplorer2, and CIRI2. They hypothesized that this circRNA modulated the expression of *miR‐12,118/FLT‐1* (*VEGFR‐1*), which was associated with inflammation and angiogenesis [25].

### 3.3 Preclinical Studies (Therapeutic Target Identification)

**FIGURE 2:**
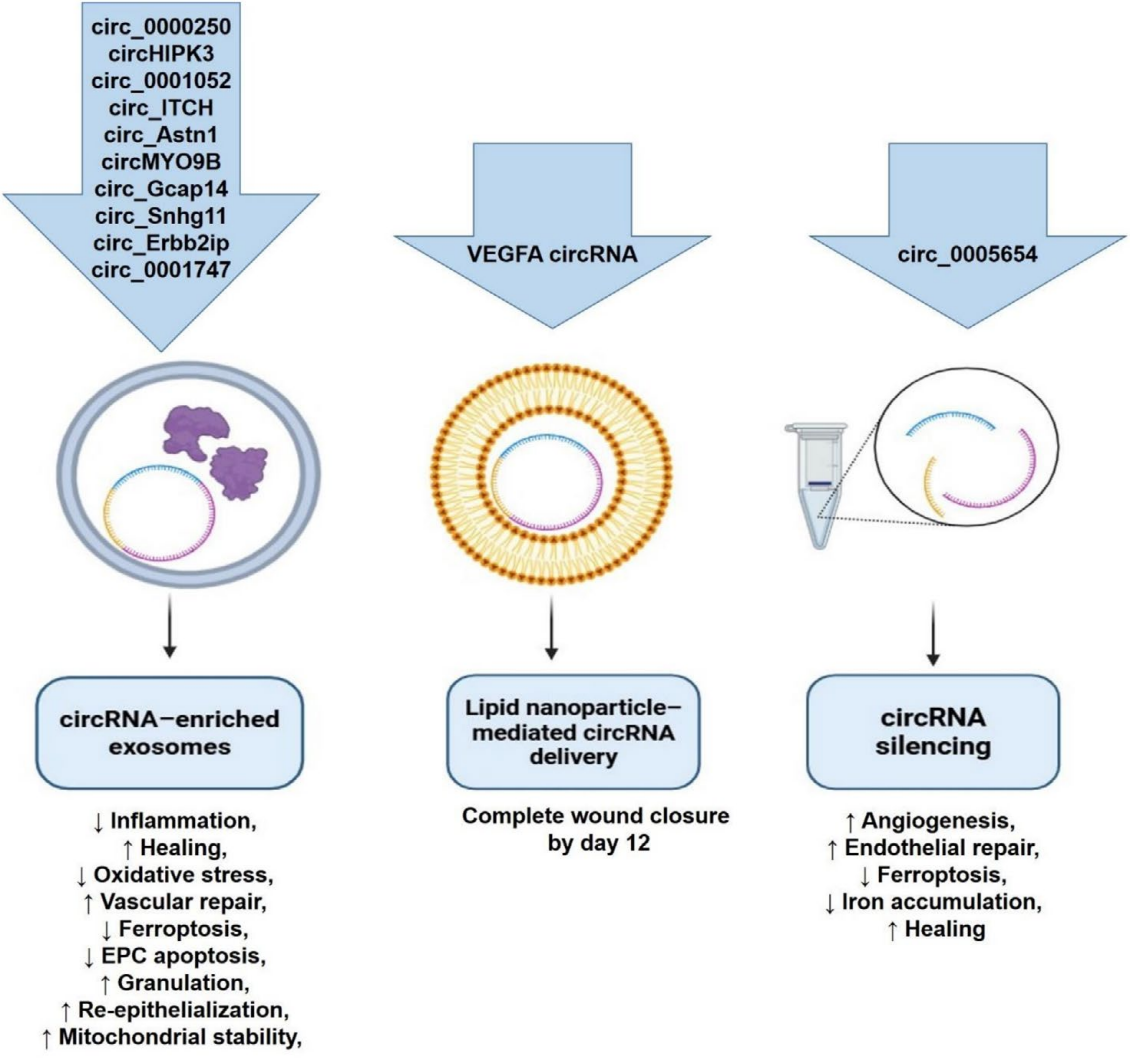
This figure summarises circRNAs identified as therapeutic or pathogenic targets in animal models of diabetic foot ulcers. Protective circRNAs delivered via exosomes, hypoxia‐preconditioned exosomes, or lipid nanoparticles promote angiogenesis, autophagy, and oxidative stress resistance. In contrast, silencing of pathogenic circRNAs such as circ_0005654 mitigates ferroptosis and inflammation.

A total of thirteen preclinical studies using mouse and rat models of DFUs were identified (Table 3) [26, 27, 28, 29, 30, 31, 32, 33, 34, 35, 36, 37, 38]. Most of the studies induced diabetes via an intraperitoneal injection of streptozotocin (STZ; 60 mg/kg, citrate buffer, pH 4.5) and confirmed hyperglycemia as a tail vein glucose level more than 250 mg/dL. Diabetic foot ulcers were typically created using 4‐mm full‐thickness punch biopsies of the hind paw or hindlimb. The reviewed studies investigated a variety of circRNA‐based interventions, broadly categorised as (i) exosome‐mediated circRNA delivery, (ii) hypoxia‐preconditioned circRNA‐enriched exosomes, (iii) nanoparticle‐assisted circRNA delivery, and (iv) circRNA silencing approaches (Figure 2).

**TABLE 3:** Summary of preclinical studies investigating CircRNAs as therapeutic targets in experimental models of DFUs.

No.	Authors (year; country)	Animal model (sex, n)	Ulcer development (wound size)	Induction of diabetes & diagnostic indicators	Intervention & intervention contents	Follow‐up/Sample collection	Main findings/Mechanistic insights
1	Shi et al. (2020; China) [26]	C57BL/6 mice (Male; *n* = 18)	4‐mm full‐thickness dorsal wound	STZ 60 mg/kg, single injection; glucose ≥ 250 mg/dL	Subcutaneous injection of ADSC exosomes (200 μg/100 μL PBS) containing mmu_circ_0000250 vs. PBS	Day 15	Exosomal *mmu_circ_0000250* promoted angiogenesis and autophagy, reduced apoptosis, and accelerated wound closure via *miR‐128‐3p*
2	Shang et al. (2021; China) [27]	C57BL mice (NA)	4‐mm full‐thickness dorsal wound	STZ 60 mg/kg; glucose ≥ 250 mg/dL	Local injection with mmu_circ_Klhl8_EPCs or SIRT5 inhibitor (MC3482, 5 mg/kg/day)	Days 0, 7, and 14	*mmu_circ_Klhl8_EPCs* restored autophagy, protected endothelial cells, and enhanced regeneration through *miR‐212‐3p/SIRT5* axis.
3	Liang et al. (2022; China) [28]	BALB/c mice (Male; *n* = 62)	Full‐thickness dorsal wound (size: NA)	STZ 200 mg/kg; glucose ≥ 250 mg/dL	UCMSC‐derived exosomes (exo‐circHIPK3) vs. exo‐vector vs. vehicle (DPBS)	Days 0, 3, 7, 14	*exo‐circHIPK3* enhanced endothelial proliferation, migration, and tube formation under hyperglycemia via *miR‐20b‐5p*
4	Wang et al. (2023; China) [29]	BALB/c mice (NA)	4‐mm full‐thickness dorsal wound	STZ 60 mg/kg; glucose ≥ 250 mg/dL	ADSC exosomes overexpressing circ_Astn1 (mmu_circ_0000101) vs. PBS (200 μg/100 μL)	Days 7, 14	*Circ_Astn1* promoted angiogenesis and inhibited EPC apoptosis via *SIRT1/FOXO1* pathway
5	Shi et al. (2022; China) [30]	C57BL/6 mice (sex: NA; *n* = 42)	4‐mm full‐thickness wound	STZ 60 mg/kg i .p.; glucose ≥ 250 mg/dL	Hypoxia‐pretreated ADSC exosomes (circ‐Snhg11) vs. PBS	Days 7, 14	Downregulation of *circ_Snhg11* promoted angiogenesis via HIF‐1α activation and M2 macrophage polarisation.
6	Wang et al. (2021; China) [31]	BALB/c mice (NA)	4‐mm full‐thickness wound	STZ 60 mg/kg; glucose ≥ 250 mg/dL	Hypoxia‐treated ADSCs vs. normoxic ADSCs	21 days	Hypoxia upregulated *circ_Gcap14*, promoting *VEGF* expression via *miR‐18a‐5p/HIF‐1α* signalling
7	Tang et al. (2024; China) [32]	C57BL/6 mice (Male; *n* = 6 per group)	4‐mm full‐thickness wound	STZ 60 mg/kg i .p.; glucose ≥ 250 mg/dL	ADSC‐Exos vs. hypoxic Exos (HExos) ± circRNAs (circ_Erbb2ip, circ_0000613, circ_0001490)	Days 7, 14	*Circ_Erbb2ip* upregulation in HExos accelerated DFU healing via *miR‐670‐5p/Nrf1* axis
8	Chen et al. (2022; China) [33]	BALB/c mice (Sex: NA; *n* = 15 per group)	Square dorsal foot wound (1 × 1 cm)	STZ 45 mg/kg × 5 days; glucose > 16.7 mM × 10 days	Diabetic BMSC‐Exos vs. OE‐circ_ITCH‐Exos (10 mg/kg, 100 μL)	Days 3, 7, 13	*Circ_ITCH* downregulated in DFU; exosomal overexpression inhibited ferroptosis and promoted angiogenesis via *TAF15*.
9	Liu et al. (2024; China) [34]	NA (Sex: NA; DFU models with 30 mice and normal mice with 12 ones)	8‐mm full‐thickness wound	High‐fat/sugar diet × 1 month + STZ i.p.; glucose > 16.7 mmol/L × 2 weeks	Topical drip of *VEGF‐A circRNA* (Unmodified‐LNP or *VEGF‐A circRNA*‐LNP)	12 days	*VEGF‐A circRNA*‐LNP achieved near‐complete regeneration by Day 12, enhancing angiogenesis.
10	Liang et al. (2022; China) [35]	BALB/c mice (male; *n* = 62)	4‐mm full‐thickness wound	STZ 45 mg/kg + high‐fat diet; glucose ≥ 16.7 mmol/L	ADSC exosomes with *circ_0001052* vs. vector vs. PBS (25 μL × 4 sites, 200 μg/100 μL PBS)	Days 3, 7, 14	*Circ_0001052* sponged *miR‐106a‐5p*, promoting *FGF4*, *VEGF*, and *p‐p38* expression to improve angiogenesis.
11	Wang et al. (2024; China) [36]	C57BLKS/J db/db diabetic mice (male; *n* = 8)	1‐cm full‐thickness wound	STZ + high‐fat diet; glucose ≥ 16.7 mmol/L	MSC‐Exos‐oe‐c*ircMYO9B* vs. sh‐*circMYO9B* vs. PBS (100 μg/100 μL)	Days 0, 3, 7, 13	*circMYO9B* promoted healing, reduced apoptosis/inflammation, and upregulated *KDM1A* and *VEGFA*
12	Li et al. (2025; China) [37]	Sprague–Dawley rats (male; *n* = 50)	2 × 5 mm rectangular dorsal wound	STZ 50 mg/kg × 5 days + high‐fat diet; glucose > 250 mg/dL	Tail‐vein injection of lentivirus (1 × 10^8^ UT/50 μL) for sh‐*circ‐0005654* ± oe‐IGF2BP2 ± Lip‐1	14 days	*Circ_0005654* promoted ferroptosis via *IGF2BP2*; silencing reduced inflammation and iron deposition
13	Wang et al. (2025; China) [38]	BALB/c mice (NA)	4‐mm full‐thickness wound	STZ 60 mg/kg; glucose > 250 mg/dL	ADSC‐Exos vs. HExos vs. *circ_0001747*_HExos vs. NC	Days 0, 7, 14	*Circ_0001747*_HExo reduced ROS and apoptosis, promoted angiogenesis via *miR‐199a‐5p/HIF‐1α* axis

#### 3.3.1 Exosome‐Mediated circRNA Delivery

A lot of research has been done on the idea that stem cell exosomes with certain circRNAs may accelerate tissue restoration in DFU models. For example, the exosomal *mmu_circ_0000250* reduced local inflammation by sequestering the pro‐inflammatory *miR‐128‐3p*. This led to the activation of *Sirtuin‐1* (*SIRT1*) and *LC3*, thereby facilitating autophagy and angiogenesis [26]. Similarly, *circHIPK3*‐enriched exosomes inhibited *miR‐20b‐5p*. This resulted in the mRNA upregulation of *nuclear factor erythroid 2–related factor 2* (*Nrf2*), *vascular endothelial growth factor A* (*VEGF‐A*), and *transforming growth factor beta 1* (*TGF‐β1*). Mechanism‐wise, it effectively attenuated oxidative stress while promoting vascular repair and wound closure [28]. Moreover, adipose‐derived stem cell (ADSC)‐derived exosomes overexpressing *mmu_circ_0001052* demonstrated a strong pro‐healing phenotype by sequestering *miR‐106a‐5p*. This mechanism activated the fibroblast growth factor 4 (FGF4)/VEGF‐A/p‐p38 signalling cascade [35]. Simultaneously, the downregulation of *mmu_circ‐ITCH* was reinstated in DFU tissues using exosomal delivery. This intervention improved angiogenesis in human umbilical vein endothelial cells (HUVECs) via activating the Nrf2 and TAF15 signalling pathways [33]. Additionally, *circ‐Astn1*‐overexpressing ADSC exosomes protected endothelial progenitor cells (EPCs) from apoptosis under hyperglycemic conditions. This was attributed to the inhibition of *miR‐138‐5p*. This was able to upregulate *SIRT1* and *Forkhead box O1* (*FOXO1*), thereby facilitating angiogenesis and epidermal regeneration [29]. Mesenchymal stem cell (MSC)‐derived exosomes containing *circMYO9B* dramatically improved wound healing by inhibiting *CBL* and increasing *Lysine‐specific demethylase 1A* (*KDM1A*) and *VEGF‐A* levels. This allowed new blood vessels to grow, granulation tissue to develop, and the epithelium to heal. In contrast, inhibiting *circMYO9B* negated these beneficial effects [36].

#### 3.3.2 Hypoxia‐Preconditioned circRNA–Enriched Exosomes

Preconditioning stem cells with hypoxia has been shown to be a significant enhancer of the effectiveness of circRNA‐mediated exosomal therapies. For instance, *mmu_circ‐Gcap14*, which was increased in ADSCs during hypoxia, was shown to block *miR‐18a‐5p*. This process augmented *hypoxia‐inducible factor 1 alpha* (*HIF‐1α*) and *VEGF* levels, which together promote angiogenesis in DFU models [31]. Similarly, *mmu_circ‐Snhg11*, which was downregulated under hyperglycemia, was restored by hypoxic exosome treatment. This restoration increased *HIF‐1α* and induced M2 macrophage polarisation through inhibition of *miR‐144‐3p*, facilitating *VEGF*‐mediated angiogenesis [30]. It was demonstrated that hypoxia exposure modified exosomal circRNA profiles by diminishing the expression of *mmu_circ‐0000495* while augmenting *mmu‐circ‐0000495, mmu‐circ‐0000613*, and *mmu‐circ‐0001490*. They collectively inhibited *miR‐670‐5p* and enhanced *Nrf1* expression, resulting in enhanced oxidative resistance and tissue regeneration [32]. Similarly, *mmu_circ‐0001747* was found to be highly expressed in hypoxia‐preconditioned ADSC‐derived exosomes and promoted wound healing through *miR‐199a‐5p*/*HIF‐1α* signalling, leading to reduced oxidative stress, apoptosis, and improved angiogenesis [38].

#### 3.3.3 Lipid Nanoparticle (LNP)–Mediated circRNA Delivery

Targeted molecular delivery via nanoparticles has also been studied. For instance, it was examined by Liu et al. [34] how VEFG‐A circRNA transported by lipid nanoparticles performed therapeutically. Topically applied VEFG‐A circRNA‐modified (A‐LNP) or unmodified (U‐LNP) nanoparticles were used by the researchers to treat animal models of DFUs. All wounds in the group treated with A‐LNP had completely healed by the 12th day [34].

#### 3.3.4 circRNA Silencing Approaches

Several studies have also shown pathogenic circRNAs that, when blocked, help wound healing. For example, overexpressing *mmu_circ‐Klhl8* in EPCs stopped *miR‐212‐3p*, which in turn increased *SIRT5*, started autophagy, and encouraged angiogenesis [27]. However, silencing *mmu_circ_0005654*, a pathogenic circRNA, markedly decreased iron buildup, inflammatory infiltration, and oxidative damage. This mechanism was accomplished by downregulating *insulin‐like growth factor 2 mRNA‐binding protein 2* (*IGF2BP2*). Thanks to this alternation, the expressions of *glutathione peroxidase 4* (*GPX4*) and *solute carrier family 7 member 11* (*SLC7A11*), markers of ferroptosis resistance, were restored, while inflammatory cytokines (*IL‐1β*, *TNF‐α*, *IL‐6*) were suppressed. The overexpression of *IGF2BP2* counteracted these effects, therefore validating the regulatory role of the *circ_0005654*/*IGF2BP2* axis in the aetiology of DFUs [37].

### 3.4 In‐Silico Studies (Candidate Discovery)

To further understand the pathophysiology of DFU, in silico investigations have shed light on the regulatory interaction networks underlying circRNA, miRNA, and mRNA. According to Table 4, two important bioinformatics research studies were found in this regard [39, 40].

**TABLE 4:** Summary of in silico studies identifying candidate CircRNAs and regulatory networks associated with DFUs.

No.	Authors (year; country)	Dataset(s)	circRNAs/miRNAs	Major findings
1	Liao et al. (2020; China) [39]	GSE114248 (circRNA), GSE84971 (miRNA), HMDD v3.0	*circ‐0089761/miR‐146a‐5p*	Identified top DFU biomarkers (AUC > 0.8): *BCL2, CCND1, IRAK4, SMAD4, SP1, SUFU. SMAD4–miR‐146a‐5p–circ‐0089761* axis linked to inflammation
2	Zeng et al. (2022; China) [40]	GSE114248, GSE84971, GSE68185, GSE80178	*hsa‐circ‐0049271, hsa‐circ‐0074559/hsa‐miR‐24‐3p, hsa‐miR‐214‐3p*	Key DFU axes: *circ‐0049271–miR‐24‐3p–JUNB*; enriched in VEGF and T‐cell signalling; associated with IL‐6–mediated inflammation and angiogenesis

Liao et al. [39] merged circRNA (GSE114248) and miRNA (GSE84971) expression datasets with diabetes‐related miRNAs from the Human MicroRNA Disease Database (HMDD v3.0). Their differential expression revealed *hsa_circ‐0089761* as a key circRNA anticipated to control inflammation‐associated *miR‐146a‐5p*. Following Receiver operating characteristic (ROC) and Gene Ontology (GO) semantic similarity studies, *BCL2, CCND1, IRAK4, SMAD4, SP1*, and *SUFU* were identified as promising biomarkers with good discriminating power (AUC > 0.8). *SMAD4, SUFU*, and *IRAK4* showed 100% sensitivity and specificity in distinguishing DFU diabetic tissues from non‐DFU diabetic tissues. *SMAD4* was the best biomarker associated with the *hsa_circ‐0089761/miR‐146a‐5p* regulatory axis, implicating this pathway in DFU‐associated inflammatory and apoptotic responses [39].

Zeng et al. [40] evaluated the GSE114248, GSE84971, GSE68185, and GSE80178 transcriptome datasets to investigate circRNA, miRNA, and mRNA co‐expression networks in DFU, diabetic (non‐DFU) tissues, and healthy tissues. They found that *hsa‐circ‐0049271* and *hsa‐circ‐0074559* were linked to the growth of DFUs. Functional enrichment tests demonstrated that the VEGF receptor and T cell signalling pathways exhibited differences in DFU tissues compared to other groups. Particularly, the *hsa‐circ‐0049271/hsa‐miR‐24‐3p/JUNB* axis regulated infectious inflammation and angiogenesis during diabetic wound development [40].

## 4 Discussion

**FIGURE 3:**
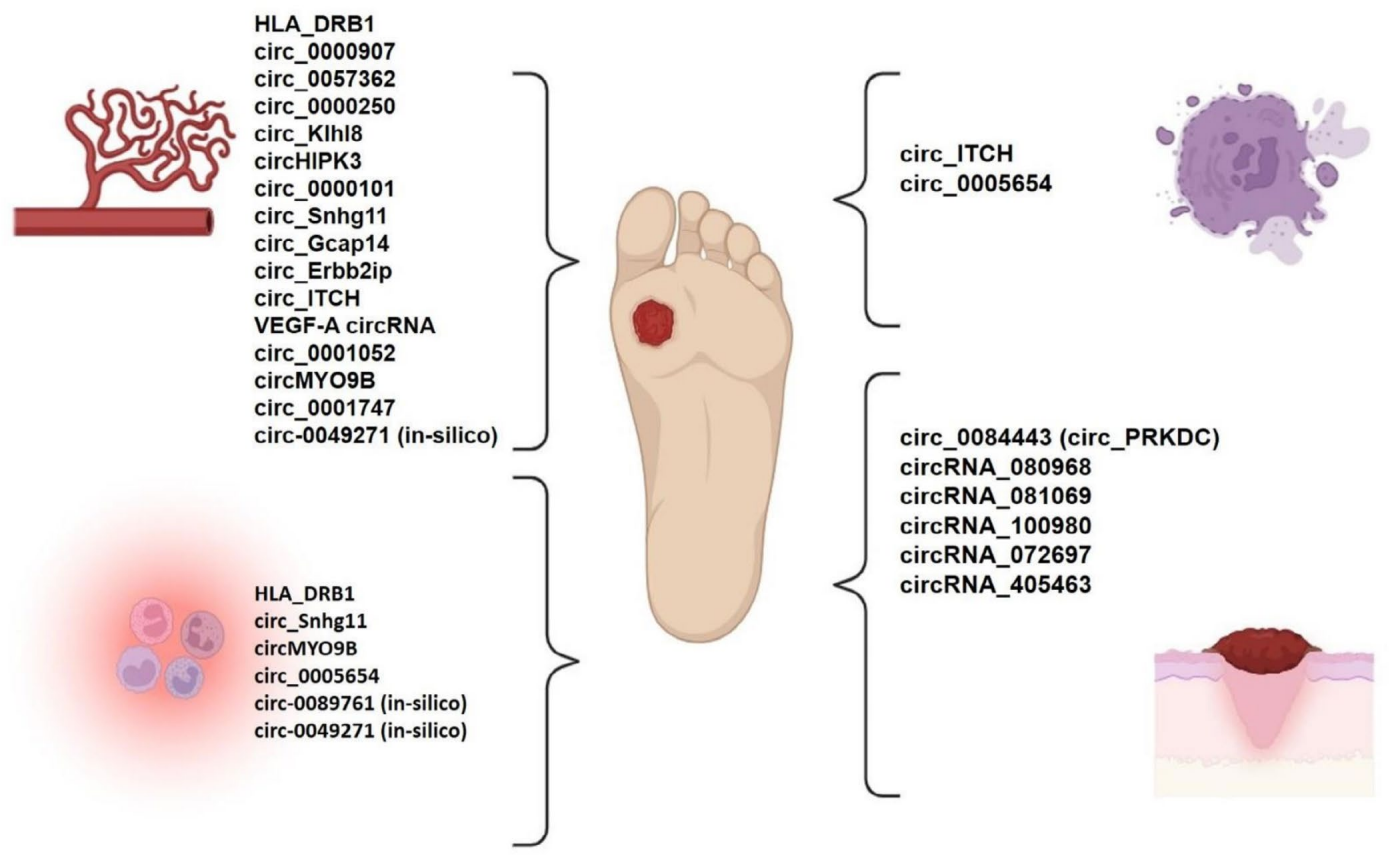
This figure depicts circRNAs identified in clinical, preclinical, and in silico studies of diabetic foot ulcers, categorised based on their role in four key biological processes: Angiogenesis, inflammation, ferroptosis/oxidative stress, and fibroblast‐keratinocyte communication/re‐epithelialization.

This study comprehensively examines the relationship between clinical, preclinical, and computational findings on the regulatory role of circRNAs in DFUs. By constructing circRNA‐miRNA‐mRNA interaction networks, we identified specific groups of circRNAs that modulate four relevant and essential biological processes for wound healing: angiogenesis, inflammation, oxidative stress response, and fibroblast‐keratinocyte communication (Figure 3). The expression levels of circRNAs, whether increased or decreased, suggest that these molecules may have both detrimental and beneficial effects on the development of DFUss.

### 4.1 Angiogenesis and circRNA Regulation

**FIGURE 4:**
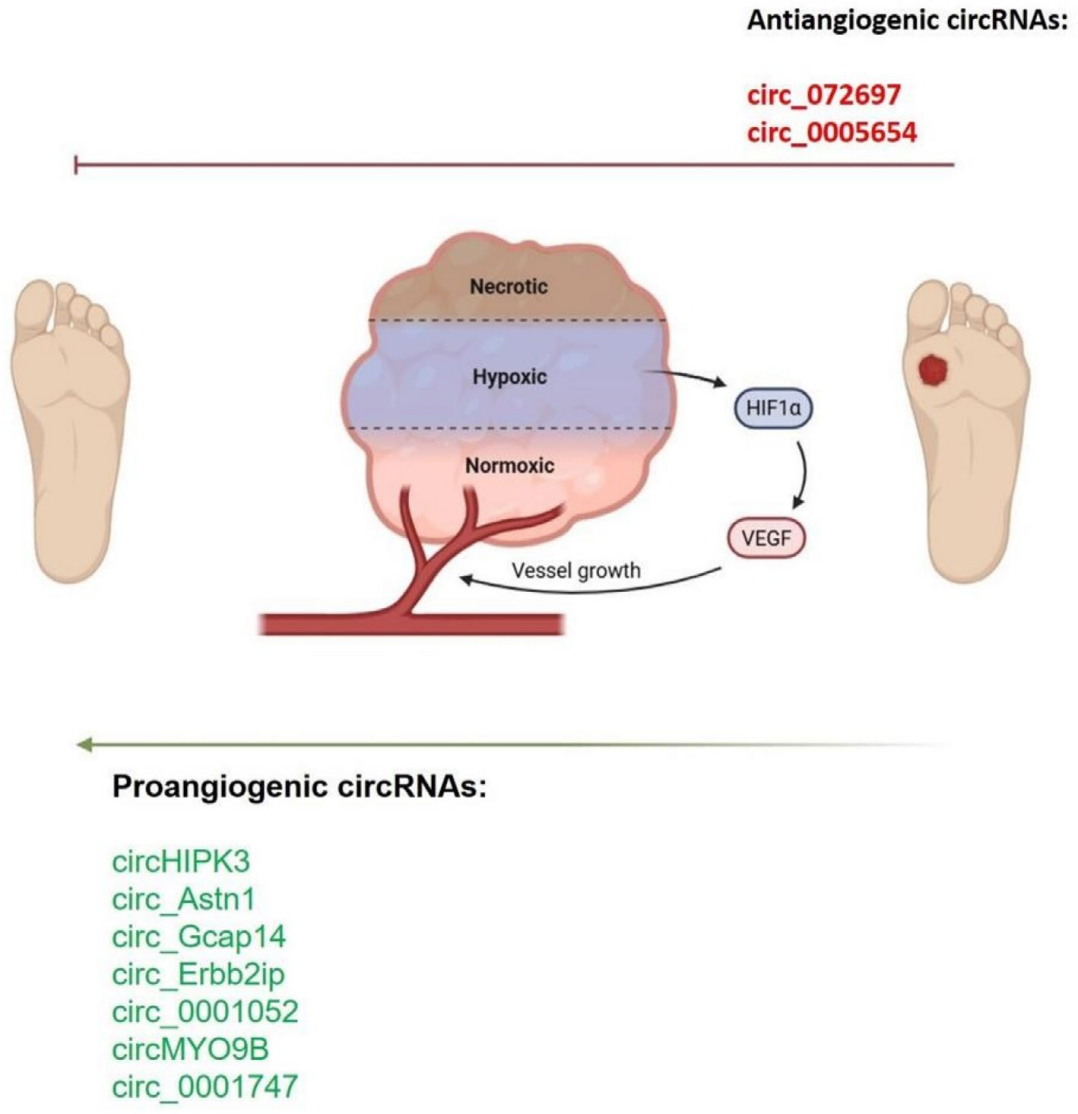
CircRNAs that either promote or inhibit angiogenesis in DFUs through regulation of HIF‐1α and VEGF‐related pathways.

Impaired angiogenesis is implicated in the development of DFUs, particularly through pathways involving HIF‐1α and VEGF (Figure 4). Hypoxia makes HIF‐1α more unstable and rapidly degraded by actions that are dependent on the prolyl hydroxylase (PHD) domain [41]. Increased methylglyoxal (MGO) in diabetes makes it more difficult for HIF‐1α to dimerize and simultaneously bind to the co‐activator (p300) [41, 42, 43]. This is due to its inhibition of the carboxyl‐terminus of Hsp70‐interacting protein (CHIP)‐mediated ubiquitination of HIF‐1α [42]. This reduces the expression of HIF‐1α target genes, including *VEGF, GLUT1*, and *EPO*, which slows wound healing and inhibits the growth of new blood vessels [41].

Our study identified several proangiogenic circRNAs that work possibly through this mechanism. They include *circHIPK3, circ_Astn1, circ_Gcap14, circ_Erbb2ip, circ_0001052, circMYO9B*, and *circ_0001747* [28, 29, 31, 35, 36, 38]. These molecules help endothelial cells grow, move, and form tubes. They also restore angiogenesis through the *miR‐20b‐5p/VEGF‐A*, *miR‐18a‐5p/HIF‐1α*, and *miR‐670‐5p/Nrf1* pathways [28, 31, 32]. *Circ_Astn1* and *circMYO9B* increased *VEGF‐A* by activating *SIRT1* and *KDM1A*, which in turn accelerated wound healing and increased blood flow in diabetic mice [29, 36].

In contrast, some circRNAs were found to inhibit angiogenesis or promote cell death. For example, *circ_072697* suppresses endothelial activity through the *miR‐3150a‐3p/KRAS–MAPK* axis [21, 22], while *circ_0005654* induces ferroptosis, a type of cell death dependent on iron accumulation and inflammation, through mechanisms mediated by *IGF2BP2* [37]. Taken together, DFUs result in impaired angiogenesis by loss of proangiogenic circRNAs and overexpression of inhibitory circRNAs. Therapeutically, increasing proangiogenic circRNAs or blocking ferroptosis‐related circRNAs may help restore vascular homeostasis and promote wound healing.

### 4.2 Oxidative Stress and Ferroptosis

**FIGURE 5:**
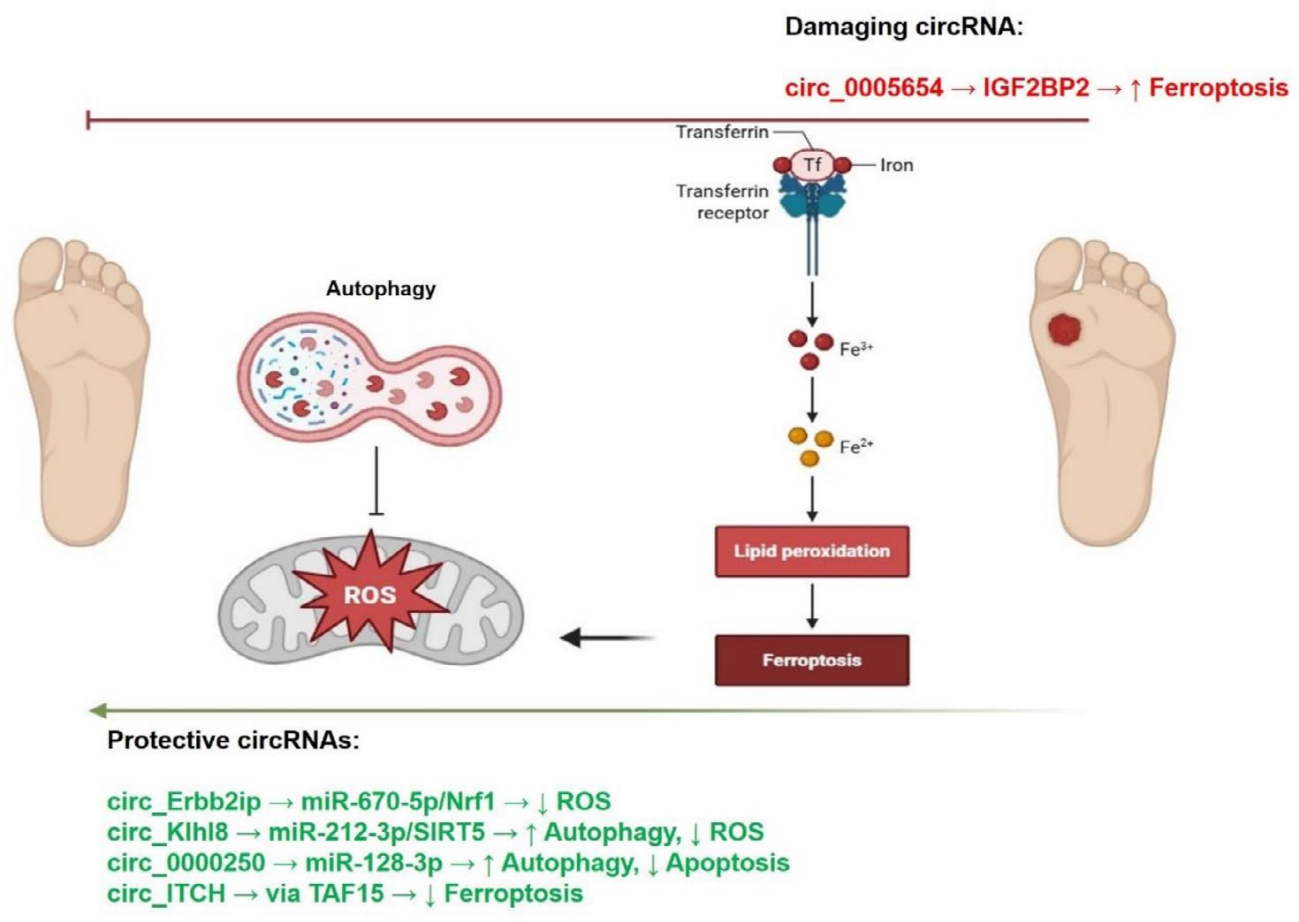
CircRNAs involved in the regulation of oxidative stress and ferroptosis in diabetic foot ulcers. Protective circRNAs increase mitochondrial stability, enhance autophagy, reduce reactive oxygen species (ROS), inhibit ferroptosis, and support angiogenesis. In contrast, *circ_0005654* acts as a deleterious circRNA by promoting ferroptosis and iron accumulation, exacerbating oxidative damage and impairing wound healing.

Chronic hyperglycemia increases the production of reactive oxygen species (ROS) and mitochondrial dysfunction, leading to ferroptosis cell death (Figure 5) [37]. However, several circRNAs help cells deal with oxidative stress. For example, *circ_Erbb2ip* and *circ_Klhl8* enhance mitochondrial stability and antioxidant defence via the *miR‐670‐5p/Nrf1* and *miR‐212‐3p/SIRT5* pathways, respectively [27]. *Circ_0000250* also stimulates autophagy via *miR‐128‐3p*, which accelerates angiogenesis and decelerates apoptosis [26]. In contrast, *circ_0005654*, which is more active in diabetic tissues, increases oxidative damage and ferroptosis more severely [37]. Exosomal *circ_ITCH*, however, which is sustained by TAF15, inhibits these effects by preventing ferroptosis and facilitating the regeneration of blood vessels [33]. These findings indicate that the exosome‐mediated production of beneficial circRNAs may represent an innovative approach to mitigate oxidative damage in this condition [26, 28, 29, 30, 31, 32, 33, 35, 36, 38].

*Nrf2*, which regulates antioxidant enzymes, is a crucial link between oxidative stress and angiogenesis [44]. In DFUs, *Nrf2* levels are significantly reduced, partly due to aberrant *histone deacetylase* (HDAC) signalling and altered DNA methylation [45]. Decreased *Nrf2, VEGF, HIF‐1α*, and *SDF‐1α*, along with increased *TSP‐2*, indicate a disruption in the redox‐angiogenic balance. Furthermore, differences in *HDAC* and *SIRT* expression (with *HDAC4* showing an inverse correlation with *Nrf2*) indicate epigenetic remodelling of DFU tissue. This *HDAC4‐Nrf2*‐angiogenesis axis may be a critical target for future therapies [45].

Iron overload exacerbates oxidative stress and inflammation, worsening tissue necrosis and vascular damage [46, 47]. Iron accumulation may also cause diabetic peripheral neuropathy, which can cause loss of sensation in DFUs [6]. Ferroptosis also induces axonal degeneration and nerve dysfunction in diabetic animal models [48]. However, therapy with liproxstatin‐1 sped up wound healing by stopping ferroptosis and inflammation [37]. These results show that circRNA‐controlled ferroptosis, oxidative stress, and angiogenesis are all closely related. This forms a molecular trio that might lead to new ways to treat persistent wounds that won't heal.

### 4.3 Inflammation‐Associated circRNA Networks

Inflammation plays a dual role in DFU, initially protective but becoming detrimental when it becomes chronic [49]. CircRNAs help fine‐tune this balance. For example, *circ_HLA‐DRB1* is higher in DFU and helps the immune system work better and change the shape of blood vessels via the *miR‐12,118/FLT‐1* axis [25]. FLT‐1, a receptor for VEGF, is very important for angiogenesis when there is inflammation. Clinical studies show that patients with DFU have higher levels of sFlt‐1, advanced oxidation protein products (AOPPs), malondialdehyde (MDA), TNF‐α, and VEGF, indicating a disruption in the angiogenesis‐inflammation interface [50]. Under these conditions, neutrophils are the main producers of VEGF and induce *FLT‐1* expression, suggesting that hyperglycemia disrupts the *VEGF/FLT‐1* balance [51]. Similarly, mast cell activation and increased *FLT‐1* have been associated with impaired angiogenesis and tissue viability in diabetic mice [52].

Meanwhile, *circ_Snhg11* increases HIF‐1α expression and promotes macrophage polarisation from proinflammatory (M1) to repair (M2), supporting inflammation resolution and matrix remodelling [30]. In contrast, *circ_0089761* and *circ_0049271* may worsen inflammation by silencing *miR‐146a‐5p* and *miR‐24‐3p* and disrupting genes such as *SMAD4, BCL2, CCND1, IRAK4*, and *JUNB* [39]. Among these, *SMAD4*, a central mediator of the TGF‐β/Smad pathway, drives M2 macrophage activation, ECM synthesis, and wound closure [53]. Thus, the circRNA‐miRNA‐TGF‐β axis emerges as a critical mechanism in regulating inflammation and tissue remodelling in DFU.

### 4.4 Fibroblast–Keratinocyte Communication and Re‐Epithelialization

For re‐epithelialization to occur properly, fibroblasts and keratinocytes must work together well. In diabetic wounds, disruption of this interaction leads to impaired wound closure [54]. Recent studies suggest that circRNAs have a regulatory function in this process. In DFUs, increased levels of *hsa_circ_PRKDC* and *hsa_circRNA_080968* can increase keratinocyte activity through the *miR‐31/FBN1/MMP2/MMP9* and *miR‐326/miR‐766‐3p* pathways [19, 20, 24]. This also activates downstream signalling pathways such as JAK/STAT, EGFR, PI3K/ERK, and HIF‐1. This leads to successful re‐epithelialization and matrix remodelling [19, 20, 24]. The ECM protein fibronectin is also crucial in this process [55]. Its continuous presence supports the entry of latent TGF‐β binding protein 1 (LTBP‐1) and latent TGF‐β into the ECM and enables fibroblast‐to‐myofibroblast differentiation through TGF‐β1‐dependent signalling [56]. The EDA domain in FN enhances LTBP‐1 binding and is essential for the acquisition of the contractile phenotype of myofibroblasts, which is essential for tissue regeneration [57, 58]. However, chronic hyperglycemia in diabetes induces excessive production of ROS, which leads to fragmentation of FN and the formation of AGEs [55, 59]. These changes not only disrupt fibroblast adhesion and morphology, but also induce sustained inflammation by stimulating neutrophil recruitment and macrophage activation [60]. The formation of FN fragments is induced in diabetic conditions, which, unlike intact FN, increases the release of MMPs and the production of inflammatory cytokines [61]. As a result, the ECM in diabetic wounds is not properly organised, making it difficult for cells to move and for blood vessels to deform [57, 58].

In addition, the researchers found that *circ_072697*, one of the downregulated circRNAs, suppresses keratinocyte growth by exploiting the *miR‐3150a‐3p/KDM2A/KRAS* pathway [21, 22]. If this regulatory circuit is disrupted, keratinocyte growth can become more difficult, and the wound matrix may not be sufficiently stable [21, 22]. The *KDM2A* gene encodes an F‐box protein that acts as a histone demethylase. This means that it plays a role in managing metabolism and inflammation [21]. Tanaka et al. [62] showed that metformin activates *KDM2A* through AMPK signalling, which prevents rapid cell growth. *KDM2A* is also involved in the inflammatory responses of keratinocytes in psoriasis [63]. This means that if it does not work properly, it may worsen inflammation in DFU and make it more difficult for epithelial cells to regrow [21]. However, the precise molecular mechanisms of *KDM2A*‐mediated circRNA regulation in diabetic wounds require further elucidation.

### 4.5 Methodological Limitations

Despite the growing interest, current evidence remains limited by several factors. First, small sample sizes and the lack of longitudinal designs in clinical studies limit generalizability and causal inference. Second, methodological heterogeneity, such as varying biological sample sources in terms of age, sex, and race, normalisation pipelines, and validation platforms, complicate cross‐study comparisons. Third, no randomised clinical trials (RCTs) evaluating circRNA‐based interventions were identified, emphasising the early stage of development of this field. Integrating a multi‐omics approach is essential to elucidate broader regulatory networks involving circRNAs.

Preclinical studies have shown promising results for the therapeutic application of exosome‐ or nanoparticle‐mediated circRNA delivery. However, several translational challenges persist. They include immunogenicity concerns, difficulties in large‐scale production, and the absence of Good Manufacturing Practice (GMP)‐grade circRNA formulations suitable for clinical use.

In silico network analyses have complemented experimental data by identifying key regulatory circuits; nonetheless, a considerable gap remains between computational predictions and experimental validation. Most bioinformatic predictions of circRNA‐miRNA interactions have yet to be validated in vivo. To fill this gap, future studies should use RNA immunoprecipitation (RIP) methods and dual‐luciferase reporter assays in DFU tissue models to validate the predicted interactions.

### 4.6 Future Perspectives

The goal of future research should be to make the ways that circRNA is isolated and profiled more consistent and standardised. This helps improve the reproducibility and repeatability of results. When organising future clinical trials, it is highly crucial to carefully consider the genetic, clinical, and social aspects of the persons who will be researched. This strategy makes it easier to find biomarkers that are effective for many people.

Advanced bioinformatics, machine learning algorithms, and deep neural networks could also help build accurate and customizable models that can help doctors diagnose DFUs early, track their progression, and monitor patient response. These kinds of advances could usher in a new era of guided tissue regeneration and precision medicine.

## 5 Conclusion

This review provides significant evidence for the essential role of circRNAs in the aetiology of diabetic foot ulcers. In silico, preclinical, and clinical studies have identified various circRNAs as potential biomarkers for disease severity and therapeutic targets, showing encouraging results in modulating angiogenesis, inflammation, oxidative stress, and fibroblast‐keratinocyte communication through circRNA‐based therapies (Figure 3). Three innovative approaches to targeting DFUs include the use of circRNA‐based nanoparticles, circRNA‐enriched extracellular vesicles, and gene silencing techniques (Figure 2), which provide a summary of circRNAs that have been used for therapeutic purposes in animal models so far. However, further research is required to convert these molecular findings into a clinically viable approach.

## Author Contributions

**A.R.G.:** writing – original draft, investigation, methodology. **M.H.:** writing – original draft; **A.S.M.:** searching, visualisation; **M.D.:** editing the manuscript, data curation; **H.C.:** writing – review and editing, conceptualization; **F.N.:** writing – review and editing, supervision, conceptualization. **R.A.:** writing – review and editing, supervision, conceptualization and methodology.

## Funding

The authors have nothing to report.

## Ethics Statement

The authors have nothing to report.

## Consent

The authors have nothing to report.

## Conflicts of Interest

The authors declare no conflicts of interest.

## Data Availability

The data that supports the findings of this study are available in the Supporting information of this article.
